# Loss of Mammographic Tissue Homeostasis in Invasive Lobular and Ductal Breast Carcinomas vs. Benign Lesions

**DOI:** 10.3389/fphys.2021.660883

**Published:** 2021-05-05

**Authors:** Evgeniya Gerasimova-Chechkina, Brian C. Toner, Kendra A. Batchelder, Basel White, Genrietta Freynd, Igor Antipev, Alain Arneodo, Andre Khalil

**Affiliations:** ^1^Laboratory of Physical Foundations of Strength, Institute of Continuous Media Mechanics UB RAS, Perm, Russia; ^2^CompuMAINE Laboratory, University of Maine, Orono, ME, United States; ^3^Department of Pathology, Perm State Medical University Named After Academician E. A. Wagner, Perm, Russia; ^4^Laboratoire Ondes et Matière d’Aquitaine, Universite de Bordeaux, Bordeaux, France; ^5^Department of Chemical and Biomedical Engineering, University of Maine, Orono, ME, United States

**Keywords:** Radiomics, tissue homeostasis, mammography, wavelets, breast density, fractals, multifractals, Hurst exponent (H)

## Abstract

The 2D wavelet transform modulus maxima (WTMM) method is used to perform a comparison of the spatial fluctuations of mammographic breast tissue from patients with invasive lobular carcinoma, those with invasive ductal carcinoma, and those with benign lesions. We follow a procedure developed and validated in a previous study, in which a sliding window protocol is used to analyze thousands of small subregions in a given mammogram. These subregions are categorized according to their Hurst exponent values (*H*): fatty tissue (*H* ≤ 0.45), dense tissue (*H* ≥ 0.55), and disrupted tissue potentially linked with tumor-associated loss of homeostasis (0.45 < *H* < 0.55). Following this categorization scheme, we compare the mammographic tissue composition of the breasts. First, we show that cancerous breasts are significantly different than breasts with a benign lesion (*p*-value ∼ 0.002). Second, the asymmetry between a patient’s cancerous breast and its contralateral counterpart, when compared to the asymmetry from patients with benign lesions, is also statistically significant (*p*-value ∼ 0.006). And finally, we show that lobular and ductal cancerous breasts show similar levels of disruption and similar levels of asymmetry. This study demonstrates reproducibility of the WTMM sliding-window approach to help detect and characterize tumor-associated breast tissue disruption from standard mammography. It also shows promise to help with the detection lobular lesions that typically go undetected via standard screening mammography at a much higher rate than ductal lesions. Here both types are assessed similarly.

## Introduction

Breast cancer is the second-most occurring cancer type, and is ranked as the fifth in terms of mortality (Siegel et al., 2015; Bray et al., 2018). The “gold standard” for assessing the state of the breast is X-ray screening mammography (Lannin and Wang, 2017). The primary and basic radiographic signs of breast cancer are masses and microcalcifications. Microcalcifications are indicative of the presence of calcium oxalate and calcium phosphate within the breast tissue, and have a small-cell character (1 mm or less in size), resembling grains of sand (Henrot et al., 2014; Wilkinson et al., 2017). Often, microcalcifications are the only radiologic manifestation of early breast cancer (Nalawade, 2009). However, this sign is not pathognomonic because in some histological forms of breast cancer, for example, lobular carcinoma, microcalcifications rarely occur (Fischmann, 2008). On the other hand, microcalcifications can occur in such benign processes as sclerosing adenosis or some fibroadenomas (Fischmann, 2008; Henrot et al., 2014). These findings are driving us to “think outside of the tumor” (Gerasimova-Chechkina et al., 2016) and to develop a computational approach to study and quantitatively characterize tissue microenvironment throughout the whole breast (Marin et al., 2017). Indeed, the breast tumor microenvironment plays a key role in early tumorigenesis. When the microenvironment is structurally sound, the tumor, if any, is under control (sleeping tumor; Bissell and Hines, 2011). If tissue structure is altered through cell cycle disruptions, the microenvironment may in fact promote tumor growth by selectively favoring the survival of cancer stem cells and protecting them from therapy or treatment (Maguer-Satta, 2011). A 2017 study presented “preliminary evidence that tissue disruption and loss of homeostasis in breast tissue microenvironment and breast bilateral asymmetry could be quantitatively and objectively assessed from mammography via a localized, wavelet-based multifractal analysis of the whole breast” (Marin et al., 2017). Tissue homeostatic balance emerges from the integration of multiple subcellular, intercellular, extracellular, chemical, and physical signals, and constraints (Rejniak, 2012). Tissue disruption is a term used in this paper and in Marin et al. (2017) to characterize what we infer as a larger-scale tissue architecture alteration caused by loss of tissue homeostasis. Contralateral asymmetry refers to imbalanced proportions of tissue organization as measured with the metrics used in this article.

The wavelet transform modulus maxima (WTMM) method is a multifractal formalism used to analyze complex 1D signals (Ivanov et al., 1999; Gerasimova et al., 2013, 2014), 2D images (Arneodo et al., 2000, 2003; Decoster et al., 2000; Roux et al., 2000; Kestener et al., 2001; Khalil et al., 2006; Batchelder et al., 2014; Marin et al., 2017), 3D images (Kestener and Arneodo, 2003), and vector fields (Kestener and Arneodo, 2004). The strategy previously developed to characterize loss of tissue homeostasis and tissue disruption (Marin et al., 2017) consists in deploying the 2D WTMM method to calculate the so-called (monofractal) Hurst exponent, *H*, by applying a sliding window approach to each mammogram. Then each subregion is color-coded based on its corresponding *H* value. Subregions where *H* ≤ 0.45 (fatty tissue) are colored blue, 0.45 < *H* < 0.55 (disrupted tissue) are yellow, and *H* ≥ 0.55 (dense tissue) are red. That pilot study demonstrated that “disrupted regions associated with loss of tissue homeostasis, as quantified by *H*∼1/2, and loss of breast symmetry, were found significantly more in tumorous cases when compared to normal cases” (Marin et al., 2017). Physical signatures associated with density fluctuations that are uncorrelated (*H*∼1/2) include randomness and free diffusion, which underpins the hypothesis that mammographic tumor-associated microenvironment displaying these uncorrelated fluctuations would be linked to a disruptive nature of the tissue and a perturbed homeostasis (Marin et al., 2017). Note that this methodology contrasts with most existing computer-aided detection/diagnostic methods (Karahaliou et al., 2008; Ayer et al., 2010; Tsai et al., 2011; Haberle et al., 2012; Meselhy Eltoukhy et al., 2012) that are predisposed for texture analysis or feature extraction.

A primary goal of this study is to demonstrate the reproducibility of the approach on a different set of mammograms than those analyzed in Marin et al. (2017). A further goal is to explore whether the tissue disruption associated with loss of homeostasis are similarly or differently characterized on patients with invasive lobular carcinomas (ILC) vs. those with invasive ductal carcinomas (IDC) [also known as invasive mammary carcinoma of no special type (Sinn and Kreipe, 2013)]. We perform a computational analysis on the mediolateral oblique (MLO) mammographic views from 81 patients with a malignant tumor (43 ILC and 38 IDC) and 23 patients with a benign tumor (12 fibroadenoma and 11 fibrocystic mastopathy).

## Materials and Methods

### The Data

Screening digital mammograms were obtained from Perm Regional Oncological Dispensary in Russia, following approval by the institution’s ethics committee. The mammographic procedure involved taking two X-ray images of the two compressed breasts for each patient, the standard MLO and craniocaudal (CC) views using an Alpha ST Mammograph (GE Healthcare). Only the MLO view was analyzed in this study. The MLO view is well-known to radiologists to include more breast tissue than any other single view (Bassett and Conner, 2003). The spatial resolution of the images is 50 μm per pixel. The data consist of mammograms with a pathology-proven diagnostic from 104 women, aged 47 to 72 (average 63.6 years old). See Table 1.

**TABLE 1:** Study design and population.

	**Histopathologic type**					
	**ILC**	**IDC1**	**IDC2**	**IDC3**	**Fib_a**	**Fib_m**
# patients	43	1	27	10	12	11
Avg age	62.3	60	64	64.3	66.5	64.2
Min age	47	60	57	61	62	60
Max age	71	60	72	70	71	69
Avg tumor size (cm)	2.1	3.7	2.2	2.5	1.7	1.3
# BN to UOQ	1	0	0	0	0	0
# CQ	4	0	1	2	2	2
# Diffuse	7	0	1	0	0	2
# LIQ	3	0	1	1	0	0
# LOQ	1	0	0	1	1	0
# LOQ LIQ border	1	0	2	0	0	0
# UIQ	3	0	3	0	0	1
# UIQ LIQ border	0	0	2	0	1	1
# UIQ to CQ	0	0	0	0	1	0
# UOQ	13	1	8	5	6	3
# UOQ LOQ border	3	0	3	0	0	0
# UOQ to UIQ	0	0	1	0	0	1
# UOQ UIQ border	7	0	5	1	1	0
# NA	0	0	0	0	0	1

*ILC, Invasive Lobular Carcinoma; IDC, Invasive Ductal Carcinoma [Invasive mammary carcinoma of no special type (Sinn and Kreipe, 2013)]; Fib_a, Fibroadenoma; Fib_m, Fibrocystic Mastopathy; BN, Behind Nipple; UOQ, Upper Outer Quadrant; CQ, Central Quadrant; LIQ, Lower Inner Quadrant; LOQ, Lower Outer Quadrant; UIQ, Upper Inner Quadrant; and UOQ, Upper Outer Quadrant.*

### WTMM Multifractal Analysis of Mammograms

The reader is referred to 2D WTMM references (Arneodo et al., 2000, 2003; Khalil et al., 2006, 2009; McAteer et al., 2010; Batchelder et al., 2014; Marin et al., 2017), specifically Marin et al. (2017), where for the first time an automated selection of critical 2D WTMM algorithmic parameters was presented. This same automated selection method was used here [see section 2.D. in Marin et al. (2017)]. A very brief overview of the 2D WTMM method follows. Using the Gaussian function θ(**r**) = exp(−**r**^2^/2), where **r**^2^ = *x*^2^ + *y*^2^, and *x*,*y* ∈ *ℝ*, the wavelet transform (WT) of a function (i.e., an image) *f*(*x*,*y*) ∈ *L*^2^(*ℝ*) with respect to the two wavelets ψ_1_(*x*,*y*) = θ(*x*,*y*)/∂⁡*x* and ψ_2_(*x*,*y*) = θ(*x*,*y*)/∂⁡*y* is the vector

$${\text{T}}_{\psi }\,\left[f\right]\,\left(b,a\right)=\nabla \left\{T_{\theta }\,\left[f\right]\,\left(b,a\right)\right\},$$

where *T*_θ_[*f*](**b**,*a*) = *a*^−2^∫∫*f*(**r**)θ(*a*^−1^(**r**−**b**))*d*^2^**r**. At any given scale *a* > 0, the WTMM are the positions **b** in the image where the WT modulus **M**_ψ_[*f*](**b**,*a*) is locally maximum. For images such as those analyzed here (i.e., everywhere continuous but nowhere differentiable rough surfaces) these WTMM form connected chains. On these chains, further maxima are found, which are themselves connected through scales to form a collection of maxima lines that are part of the so-called WT space-scale skeleton, **L**(*a*). The partition functions

$$\text{Z}\,\left(q,a\right)=\sum_{l\in L\,\left(a\right)}{\left(\underset{\left(x,a^{\prime }\right)\in L,a^{\prime }\leq a}{\sup}{\text{M}}_{\psi }\,\left[f\right]\,\left(b,a\right)\right)}^{q},$$

where *q* ∈ *ℝ* are statistical order moments, allow us to obtain the scaling exponents τ(*q*) via the relation **Z**(*q*,*a*)∼*a*^τ(*q*)^, from which we can then obtain the singularity spectrum $D\left(h\right)=\min_{q}(qh-\tau \left(q\right)$. Here *D* is the fractal dimension of the set of points in the domain of *f* where the local Holder roughness exponent is *h*, i.e., the set of all points **r** where *f*(**r**+*ϵ*)−*f*(**r**)∼*ϵ*^*h*(**r**)^,|ϵ|→0. When the Holder exponent is identical throughout the domain of *f*, then the global Hurst roughness exponent, *H* is used to characterize the monofractal scaling properties. As discussed in Marin et al. (2017), the mammogram subregions almost always display monofractal scaling properties (those that do not are discarded from the analysis), and therefore, a single Hurst exponent value is associated to each subregion.

### Image Sliding Window Analysis

**FIGURE 1:**
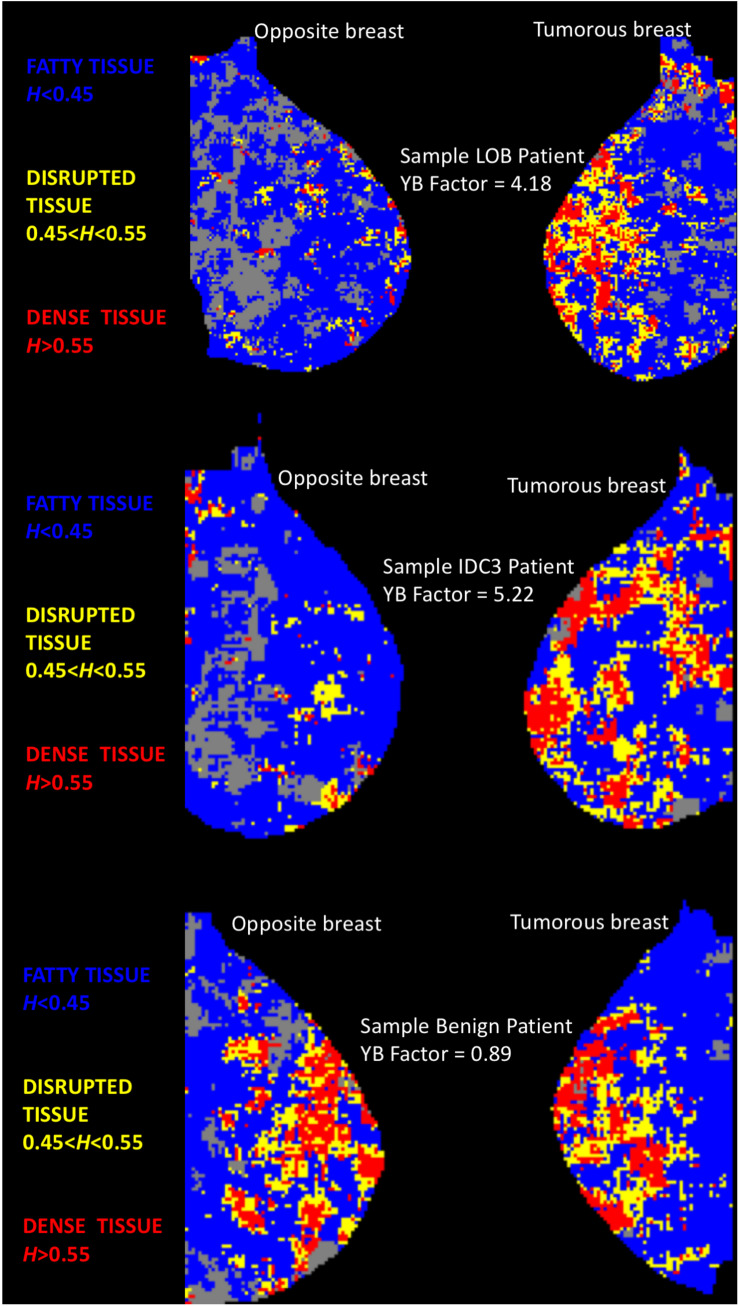
Color-coded MLO views of the tumorous and opposite breast from three sample patients. Each pixel represents “a 360 × 360-pixel mammogram subregion colored according to its *H* value. Subregions where *H* ≤ 0.45 (fatty) are colored blue, 0.45 < *H* < 0.55 (disrupted) are yellow, and *H* ≥ 0.55 (dense) are red. Gray pixels correspond to rejected subregions” [see (Marin et al., 2017)]. **TOP:** Patient with an invasive lobular carcinoma. **MIDDLE:** Patient with an invasive ductal carcinoma. **BOTTOM:** Patient with a benign lesion (fibrocystic mastopathy). For the two cancer patients, the two MLO panels show evident differences in terms of yellow (disrupted tissue, 0.45 < *H* < 0.55) subregions. However, for the benign patient, both breasts do not display evident visual differences in tissue disruption.

As was done in Marin et al. (2017), each mammogram was divided into several thousands of overlapping subregions of size 360 × 360-pixel, of which only the central 256 × 256 wavelet-transformed portion was kept for analysis, by sliding a window by 32-pixel increments, going from the top left portion of the mammogram, down to the bottom right. Then the 2D WTMM method, for which the scaling parameters were set automatically via the objective method presented in Marin et al. (2017), was used compute the Hurst exponents for each subregion, except when the automated method failed to identify scaling parameters satisfying user-inputted minimal threshold requirements, as discussed in Marin et al. (2017). These latter cases were discarded from the analysis and shown in gray in Figure 1. Otherwise, the algorithm associated a color to the value of *H*: subregions where *H* ≤ 0.45 correspond to anti-correlated density fluctuations, which were found to be associated with fatty tissue, were colored blue; subregions where *H* ≥ 0.55 correspond to long-range correlated density fluctuation, which were found to be associated with dense tissue, were red; and subregions where 0.45 < *H* < 0.55 correspond to uncorrelated density fluctuation, which we refer to as disrupted tissue, were yellow (Marin et al., 2017). Sample cases are presented in Figure 1.

### Metrics

**FIGURE 2:**
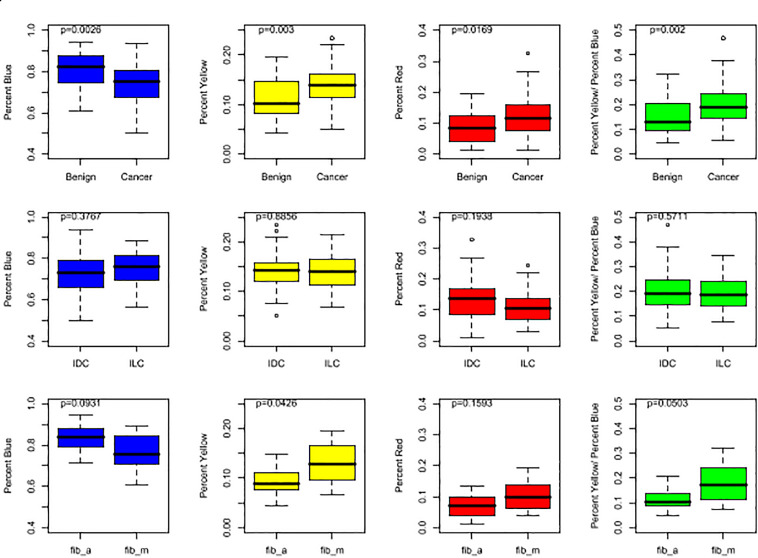
Box plots representing the distributions of %B (in blue), %Y (in yellow), %R (in red), and %Y/%B (in green). For the top row, all cases are included for both cancer and benign patients. In the middle row, the IDC population is compared to the ILC population. In the bottom row, the two benign subgroups, fib_a and fib_m, are compared. The *p*-values shown at the top of each plot were obtained by running a non-parametric Wilcoxon Rank Sum test.

The following metrics were calculated for each mammogram: the percentage of blue subregions per mammogram (%B), the percentage of yellow subregions per mammogram (%Y), the percentage of red subregions per mammogram (%R), and a combination score: %Y/%B. The latter metric was implemented based on empirical evidence that tumorous breasts had not only higher yellow subregions concentrations, but also lower blue subregions concentrations (Figure 2). And finally, to measure similarity between the tumorous breast and its contralateral counterpart, another metric, coined “YB Factor,” calculates the ratio of the %Y/%B scores from the tumorous breast to the contralateral opposite:

$$\mathrm{YB}\,\mathrm{Factor}=\,\frac{\%Y_{\text{tumor}}/\%B_{\text{tumor}}}{\%Y_{\text{opposite}}/\%B_{\text{opposite}}}.$$

The introduction of the YB Factor brings two advantages. It yields a single score per patient, which allows us to perform population statistics on patients (in addition to statistics on populations of breasts). And since the YB Factor is a ratio, its value is meaningful: YB Factor ∼ 1 is representative of similarity between the two breasts and any value away from 1 represents and quantifies asymmetry. Radiologists typically consider contralateral asymmetry as a feature of tumor development because breast asymmetry was shown to be greater in healthy women who later developed breast cancer than in women who did not (Scutt et al., 2006).

### Statistical Tests

Using the Shapiro–Wilk’s test of normality, we found that some distributions were normal and others were not (Royston, 1982). All statistical distribution analyses and Wilcoxon Rank Sum tests (yielding the unadjusted *p*-values presented in this article) were performed using the R language, version 3.6.2 (R Core-Team, 2019).

## Results

**FIGURE 3:**
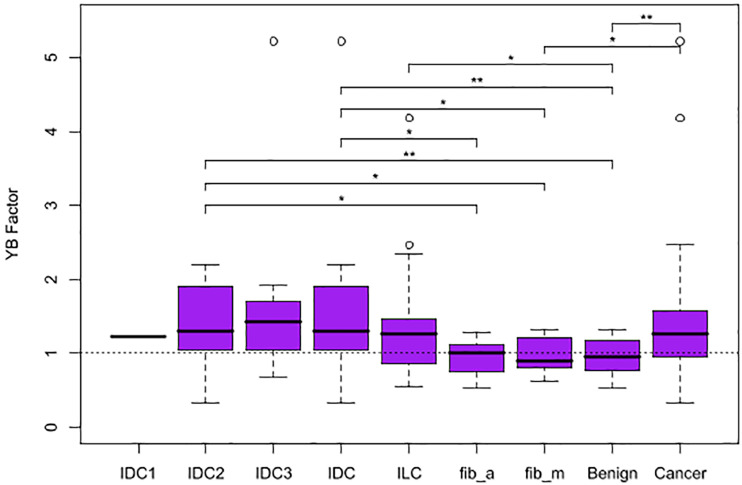
Box plots representing the distribution of YB Factor for all subgroups of patient data. A horizontal dashed line is included at YB Factor = 1, an indicator of similarity between the tumorous and the opposite breast. One star represents *p*-value < 0.05 and two stars represent *p*-value < 0.01.

The four metrics were calculated for each mammogram (Figure 2), and the YB Factors were calculated for each patient (Figure 3). Statistical significance tests were performed using the non-parametric Wilcoxon Rank Sum test and box plots were created to display the differences and similarities between different types of breast and patient populations. The box plots and *p*-values are presented in Figures 2, 3, where for the latter one star represents a *p*-value < 0.05 and two stars represents *p*-value < 0.01. In these two figures, blue represents %B, yellow represents %Y, red represents %R, green represents %Y/%B, and purple represents the YB Factor. In Figure 2 the top row represents all cancer breasts vs. all benign breasts, the middle row represents the invasive ductal carcinoma (IDC^1^) breasts vs. the ILC breasts, and the bottom row represents the two benign breast categories: fibroadenoma (fib_a) vs. fibrocystic mastopathy (fib_m). In these figures, “Cancer” refers to the combined data from both IDC and ILC and “Benign” refers to the combined data from fib_a and fib_m. In Figure 3, the IDC data are broken down into their pathological grades (IDC1, IDC2, IDC3 referring to grades 1, 2, 3, respectively, – see Table 1).

Overall, there is a statistically significant difference between all tumorous breasts with cancer (IDC and ILC combined) when compared to all breasts with a benign lesion (fib_a and fib_m combined). Cancer breasts have higher levels of disrupted tissue than benign breasts using both the %Y metric (*p*-value ∼ 0.003 – Figure 2, top yellow) and the disrupted tissue relative to fatty tissue metric, i.e., %Y/%B, (*p*-value ∼ 0.002 – Figure 2, top green).

On a patient level, when comparing breast asymmetry via the YB Factor, again, there is a statistically significant difference between the YB Factors from cancer patients vs. the YB Factors from benign patients (*p*-value ∼ 0.0062). In addition to measuring significant differences, the YB Factors yield a direct assessment of asymmetry: in terms of proportions, approximately 75% of the cancer patients had a YB Factor > 1 vs. only ∼50% for the benign cases (Figure 3).

Individually, the IDC patients and the ILC patients are significantly different than the benign patients when considering the YB Factor (Figure 3). The *p*-value for IDC vs. benign is ∼0.003 and the *p*-value for ILC vs. benign is ∼0.035. However, and quite notably, there is no significant difference between IDC breasts vs. ILC breasts.

And finally, we further note that the breasts fibrocystic mastopathy (fib_m) have higher levels of disrupted tissue than breasts with fibroadenoma (fib_a). This is true based on the %Y metric (Figure 2, bottom yellow, *p*-value ∼ 0.0426) as well as the %Y/%B metric (Figure 2, bottom green, *p*-value ∼ 0.0503). However, it is critically important to note that there is no significant asymmetry with the benign cases, as assessed by the YB Factor (Figure 3).

## Discussion

Using a previously published computational methodology to assess tumor-associated loss of homeostasis via mammography, referred to as “tissue disruption,” we performed an analysis of tumorous breasts (benign vs. cancer) as well as an analysis of patients (tumorous breast vs. the contralateral opposite). Mammograms of cancerous breasts were found to have significantly higher levels of tissue disruption compared to benign breasts. Additionally, on a patient population level, the breast disruption asymmetry, as measured here via the newly introduced YB Factor, showed that while benign patients have relatively similar disruption levels in both breasts (YB Factor ∼ 1), that ratio is significantly higher in the tumorous breast for cancer patients. This means that when comparing the tumorous breasts to the healthy opposites, only the malignant breasts are significantly different from their opposites, not the benign breasts. This not only adds obvious diagnostic value to the tumorous vs. opposite breast approach, but, through further study, may lead to a better biophysical and physiological understanding of difference in loss of tissue homeostasis in malignant vs. benign microenvironments.

Patients with IDC and those with the much rarer (but more difficult to detect) ILC were individually significantly different than the benign population. Our investigation into potential differences between IDC and ILC showed that no significant differences existed between the two populations. This leads us to hypothesize that our methodology may be particularly well-suited to characterize tissue disruption associated with ILC and not just IDC. ILC is known for being difficult to detect radiologically (Krecke and Gisvold, 1993; Brem et al., 2009; Lopez and Bassett, 2009). It is typically detected at a later stage than IDC (Chen et al., 2017) and the mammographic appearance of ILC is often dangerously subtle (Johnson et al., 2015). Although ILC only accounts for 5–10% of breast cancers (Pestalozzi et al., 2008), it has an inherently invasive growth pattern and it disproportionately has greater metastasis compared to IDC (Arpino et al., 2004; Ferlicot et al., 2004; Chen et al., 2017; Nayeem et al., 2018). Moreover, ILC is more likely to lead to mastectomy and a lower long-term (5+ years) survival rate than IDC (Pestalozzi et al., 2008; Chen et al., 2017). Therefore, further validation of our methodology on a larger cohort could eventually translate to a major advancement in earlier detection of lobular breast cancer.

Important distinctions separate the analysis performed in previous work (Marin et al., 2017) vs. what is reported here. In the former, the analysis was performed on digitized mammogram films (scans) obtained from the Digital Database for Screening Mammography (DDSM; Heath et al., 1998, 2001), while here digitally acquired mammograms were analyzed. Although the pixel sizes are similar in both studies (∼50 microns/pixel), the dynamic range is different: lossless LJPEG 12-bit data (pixel values from 0 to 4095) for the DDSM data and uncompressed 8-bit BMP data (0–255) for the current study. Nonetheless, disrupted tissue regions (yellow squares, where the Hurst exponent, *H*, is between 0.45 and 0.55) are significantly found in larger numbers in tumorous breasts in both studies. This is important for two reasons: it demonstrates reproducibility of the approach (different patient populations were used in each study), and the same method offers a similar diagnostic potential in scans *and* digitally acquired mammograms.

A more refined study will be undertaken to investigate more patients, with additional metrics [also see Marin et al. (2017)], and to explore whether or not (and to what extent) different histopathological types of tumors could be discriminated with our methodology. In particular, future investigations and explorations of combinations of metrics are recommended for larger cohorts, ideally with longitudinal datasets.

## Data Availability Statement

The raw data supporting the conclusions of this article will be made available by the authors, without undue reservation.

## Ethics Statement

The studies involving human participants were reviewed and approved by Local Ethics Committee of Perm State Medical University named after E.A. Wagner (protocol No 10 from 28/11/2018). The patients/participants provided their written informed consent to participate in this study.

## Author Contributions

EG-C, GF, and IA: data acquisition. BW: image pre-processing. BT and AK: WTMM analysis. EG-C, KB, and AK: statistical analyses and figure preparation. EG-C, KB, and AK: manuscript writing. All authors have read and approved of the manuscript.

## Conflict of Interest

We declare the United States Patent 10,467,755 *Methods of Cancer Detection* 11/05/2019. Inventors: AK and KB. The remaining authors declare that the research was conducted in the absence of any commercial or financial relationships that could be construed as a potential conflict of interest.

## Footnotes

1 Also referred to as invasive mammary carcinoma of no special type (Sinn and Kreipe, 2013).
